# Human Peroxin PEX3 Is Co‐translationally Integrated into the ER and Exits the ER in Budding Vesicles

**DOI:** 10.1111/tra.12350

**Published:** 2015-12-21

**Authors:** Peter U. Mayerhofer, Manuel Bañó‐Polo, Ismael Mingarro, Arthur E. Johnson

**Affiliations:** ^1^ Department of Molecular and Cellular MedicineTexas A&M Health Science Center440 Reynolds Medical BuildingCollege StationTX77843USA; ^2^ Institute of Biochemistry, BiocenterGoethe University FrankfurtMax‐von‐Laue Str. 960438FrankfurtGermany; ^3^ Present address: School of Biosciences & MedicineUniversity of SurreyGuildfordGU2 7XHUK; ^4^ Departament de Bioquimica i Biologia MolecularUniversitat de ValenciaC/ Dr. Moliner, 50E‐46100BurjassotSpain; ^5^ Department of ChemistryTexas A&M UniversityCollege StationTX77843USA; ^6^ Department of Biochemistry and BiophysicsTexas A&M UniversityCollege StationTX77843USA

**Keywords:** budding vesicles, endoplasmic reticulum, human peroxisomal membrane protein PEX3, peroxisomal biogenesis, Sec61 translocon

## Abstract

The long‐standing paradigm that all peroxisomal proteins are imported post‐translationally into pre‐existing peroxisomes has been challenged by the detection of peroxisomal membrane proteins (PMPs) inside the endoplasmic reticulum (ER). In mammals, the mechanisms of ER entry and exit of PMPs are completely unknown. We show that the human PMP PEX3 inserts co‐translationally into the mammalian ER via the Sec61 translocon. Photocrosslinking and fluorescence spectroscopy studies demonstrate that the N‐terminal transmembrane segment (TMS) of ribosome‐bound PEX3 is recognized by the signal recognition particle (SRP). Binding to SRP is a prerequisite for targeting of the PEX3‐containing ribosome•nascent chain complex (RNC) to the translocon, where an ordered multistep pathway integrates the nascent chain into the membrane adjacent to translocon proteins Sec61α and TRAM. This insertion of PEX3 into the ER is physiologically relevant because PEX3 then exits the ER via budding vesicles in an ATP‐dependent process. This study identifies early steps in human peroxisomal biogenesis by demonstrating sequential stages of PMP passage through the mammalian ER.

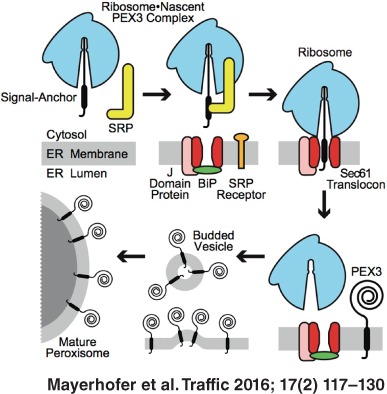

The significance of peroxisomes in cellular metabolism is illustrated by the existence of severe inherited human diseases that result from the failure of peroxisomal biogenesis 1, 2. More than 30 proteins (termed peroxins) are involved in peroxisomal assembly across species (reviewed in 3, 4, 5), but only three are key players in early peroxisomal membrane biogenesis. PEX19 is a soluble protein that acts as receptor and chaperone for newly synthesized peroxisomal membrane proteins (PMPs) in the cytosol 6. The integral PMP PEX16 mediates the endoplasmic reticulum (ER)‐to‐peroxisome trafficking of PMPs 7, 8, but homologues are absent in most yeast species 9. The PEX3 PMP is highly conserved among species and has been proposed to be the docking factor for cytosolic PEX19•cargoPMP complexes 10, 11. In yeast, PEX3 is also involved in organelle inheritance and peroxisomal autophagic degradation (pexophagy) 12, 13.

Peroxisomes have long been considered to be autonomous organelles that arise exclusively by growth and division of pre‐existing peroxisomes 14, 15. However, convincing evidence has recently shown that at least a subpopulation of PMPs in yeast 16, 17, 18, 19, 20, 21, 22, plant 23 and vertebrate cells 24, 25, 26, 27 are targeted first to the ER prior to being transported to the peroxisomes via an ER‐derived vesicle carrier 28, 29, 30. This ER‐mediated biogenesis pathway also emphasized the key roles of PEX3 and PEX19 in early peroxisomal assembly, due to their newly identified functions in intra‐ER sorting of PMPs, and the budding of preperoxisomal vesicles 19, 22, 28, 29. Eventually, the combined evidence that certain PMPs are sorted either indirectly through the ER or directly to pre‐existing peroxisomes evolved into the semiautonomous model of peroxisomal biogenesis 23, where both pathways are supposed to operate simultaneously 3, 31, 32, 33, 34.

As a prerequisite to understanding the early ER‐mediated steps in peroxisomal biogenesis, it is essential to ascertain how peroxins are targeted to and inserted into the ER membrane. For the small group of tail‐anchored PMPs, two pathways have been identified as being involved: Insertion of mammalian PEX26 is mediated by PEX19 and PEX3 35, whereas yeast tail‐anchored PMPs are most likely post‐translationally inserted via the GET3 pathway 20, 36. However, the majority of PMPs are polytopic or type I/II integral membrane proteins. In yeast, such PMPs appear to be inserted through the yeast Sec61p translocon 20, 21 that serves as the primary ER entry point for integral membrane and secretory proteins. Depending on its exact protein composition, the yeast Sec61p complex promotes co‐ and post‐translational translocation of proteins 37, 38. Which of these pathways is taken for the translocation of yeast PMPs is unknown, because previous studies 20, 21 did not reveal any mechanistic details about how the yeast Sec61p complex facilitates PMP insertion into the ER bilayer. In addition, it remains unresolved how yeast or mammalian PMPs are selected for ER insertion rather than being targeted to pre‐existing peroxisomes. Making things even more complicated is the fact that the underlying molecular mechanisms may be different in species that are evolutionarily diverse. For instance, the function and topology of a critical component in early human peroxisomal biogenesis, PEX16 39, varies between species: it is an integral membrane protein functioning as a PMP receptor in mammals 11, 25, a peripheral membrane protein involved in peroxisomal fission in *Yarrowia lipolytica* 40, and most yeast species lack a PEX16 homologue 41, 42. Hence, it is not always appropriate to extrapolate the knowledge gained from one organism to another evolutionarily diverse species 43, especially for complex mechanisms such as those that facilitate the ER targeting and insertion of PMPs.

With regard to their important role in human metabolism, surprisingly little is known about the passage of PMPs through the mammalian ER, including the identity of the translocon, if any, that facilitates PMP membrane insertion. In addition, it is not known whether ER‐derived vesicles play a role in mammalian PMP trafficking to peroxisomes, and no suitable *in vitro* system has been established to address this issue. In this study, we identify sequential stages in the co‐translational biogenesis of a human PMP, PEX3, as it enters and exits the mammalian ER. We show for the first time that the signal recognition particle (SRP) targets a peroxisomal integral membrane protein to the ER, and that PEX3 integration into the mammalian ER membrane occurs co‐translationally at the Sec61‐translocon in a multistep process. We also establish a mammalian cell‐free membrane budding assay as an experimental platform to reveal that PMP‐containing vesicles are released from the ER in an energy‐dependent reaction.

## Results and Discussion

### Approach

Convincing evidence has been provided that certain mammalian PMPs 24, 25, 27, 44, including human PEX3 26, are first targeted to the ER on route to the peroxisome. In light of its important role in early peroxisomal biogenesis 45, we have focused on identifying the mechanisms involved in human PEX3 targeting to and insertion into the mammalian ER membrane. Thus, we used an *in vitro* translation system well established for studying ER targeting 46, 47, the co‐translational SRP/translocon‐dependent targeting and integration of nascent proteins into the ER membrane 37, 38, 48, 49, and transient nascent protein interactions during translation 50.

**Figure 1:**
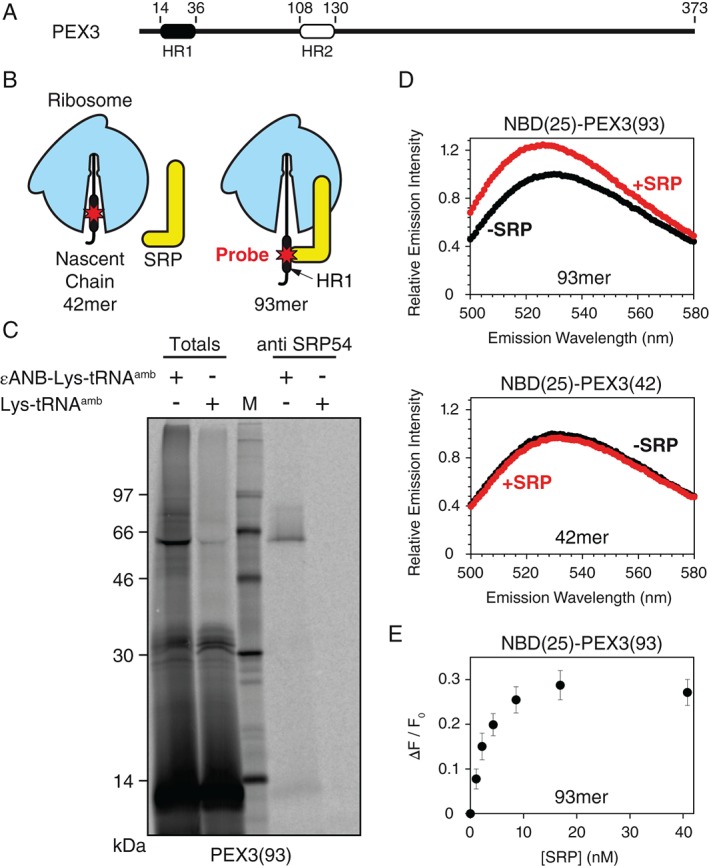
**HR1 of PEX3 binds to SRP.** A) Schematic representation of full‐length PEX3. Two predicted hydrophobic α‐helical regions are indicated by black (HR1) and white (HR2) boxes. B) A probe (the photoreactive crosslinker ANB or the fluorescent dye NBD) is incorporated into HR1 of ribosome‐tethered nascent PEX3. The HR1 of short nascent chains (e.g. 42mer) is located within the ribosomal exit tunnel, whereas longer chains (e.g. 93mer) expose HR1 to the cytosol and hence to the SRP. C) Photocrosslinking of PEX3 to SRP. [^35^S]Met‐PEX3(93)‐RNCs with or without a single ANB at residue 25 were photolyzed and then analyzed by SDS‐PAGE and phosphorimaging either directly (Totals, 1/20 aliquot) or after immunoprecipitation with antibodies directed against SRP54. M: molecular weight marker. D) Fluorescence‐detected SRP binding to PEX3. Emission scans (λ_ex_ = 468 nm) of purified NBD(25)‐PEX3(93)‐ or NBD(25)‐PEX3(42)‐RNCs were performed in buffer A before (−SRP) and immediately after the addition of purified canine SRP (+SRP). E) Purified NBD(25)‐PEX3(93)‐RNCs were titrated with the indicated total concentrations of SRP. The observed change in emission intensity (λ_ex_ = 468 nm; λ_em_ = 528 nm) is ΔF, and the initial fluorescence intensity of the sample without SRP is designated F_0_. The averages of at least three independent experiments are shown, with error bars indicating the SD.

As soon as a cleavable signal sequence or an uncleaved signal‐anchor sequence of an integral membrane protein emerges from the ribosome, it is recognized and bound by the SRP (reviewed in 37 38). This interaction transiently arrests protein synthesis until the SRP interacts with its ER‐resident receptor to target the ribosome•nascent chain complex (RNC) to a translocon in the ER membrane. Two hydrophobic regions, HR1 and HR2 (Figure 1A), have been identified in human PEX3 51. Since HR1 emerges first from the ribosomal exit tunnel during ribosomal synthesis (Figure 1B), its interactions were examined using environmentally sensitive probes. A photoreactive crosslinking probe (5‐azido‐2‐nitrobenzoyl, ANB) or a fluorescent dye (7‐nitrobenz‐2‐oxa‐1,3‐diazole, NBD) was positioned in the middle of HR1 by *in vitro* translation of a human PEX3 mRNA in which codon 25 was replaced by an amber stop codon (PEX3^G25amb^, see also Figure 3A). Addition of amber suppressor aminoacyl‐tRNA analogs ϵANB‐Lys‐tRNA^amb^ or ϵNBD‐Lys‐tRNA^amb^ 52, 53, 54 to the translation then allowed selective labeling of HR1 with the probe. When a truncated PEX3 mRNA transcript lacking a final stop codon was translated, all nascent chains in the resulting RNC sample had the same length and remained attached to the ribosome as peptidyl‐tRNA because normal termination was prevented. By varying the length of truncated mRNA added to translations, RNCs with different nascent chain lengths provided a series of static snapshots of sequential stages in PMP membrane targeting and integration. Nascent chains are designated P(*x*)‐PEX3(*n*) to represent PEX3 nascent chains with a length of *n* residues and a probe P at residue *x*. Since the ribosomal exit tunnel encloses roughly 40 residues of an emerging nascent chain, and HR1 extends until residue 36 of PEX3, a RNC with a nascent chain of approximately 80 residues is necessary to fully expose HR1 to the cytosol. Hence, initial experiments were performed with PEX3(93)‐RNCs (Figure 1B), thereby ensuring sufficient spatial flexibility and distance between HR1 and the ribosome.

### SRP binds the N‐terminal HR1 of ribosome‐bound nascent PEX3

Photoreactive ANB was introduced into HR1 by translating a truncated PEX3^G25amb^ mRNA in the presence of ϵANB‐Lys‐tRNA^amb^; the control sample received Lys‐tRNA^amb^. The resulting ANB(25)‐PEX3(93) and PEX3(93) RNCs were photolyzed, and a prominent photoadduct of approximately 65 kDa, which represents the molecular weigh of SRP54 (54 kDa) covalently linked to the PEX3 93mer (11 kDa), was formed only in the sample with ANB (Figure 1C). Photoadducts were then analyzed by immunoprecipitation using antibodies specific for SRP54, the signal sequence‐binding component of SRP 55, 56. Since [^35^S]Met‐labeled ANB(25)‐PEX3(93) chains reacted covalently with SRP54 (Figure 1C), the photoreactive ANB in HR1 was adjacent to SRP54. On the other hand, the shorter ANB(25)‐PEX3(61) RNC, which does not expose HR1 completely to the cytosol, did not form covalent photoadducts with SRP54 (Figure S1, Supporting Information). Thus, HR1 was recognized and bound by SRP as it emerged from the ribosome.

The association of SRP with PEX3‐containing RNCs was also detected using a NBD fluorescent probe in HR1. NBD was chosen because its emission properties change dramatically upon moving from an aqueous to a hydrophobic environment 52, and we previously showed that NBD was a sensitive spectral sensor of SRP association with a RNC signal sequence 53. NBD was introduced at position 25 of HR1 by translating truncated PEX3^G25amb^ mRNA in the presence of ϵNBD‐Lys‐tRNA^amb^. When canine SRP was added to purified NBD(25)‐PEX3(93)‐RNCs, a significant increase in NBD emission intensity was observed (Figure 1D, top). In contrast, no increase in emission intensity was detected when only buffer was added to NBD(25)‐PEX3(93) RNCs (Figure S2) or when SRP was incubated with NBD(25)‐PEX3(42) RNCs (Figure 1D, bottom) with HR1 still inside the ribosomal exit tunnel (Figure 1B). Moreover, SRP binding to NBD(25)‐PEX3(93)‐RNCs was saturable, as shown by the dependence of sample emission intensity on the concentration of SRP (Figure 1E). These data therefore provide the first direct evidence that a nascent peroxisomal integral membrane protein is recognized and bound by the SRP as soon as it emerges form the ribosomal exit tunnel.

### HR1 functions as signal‐anchor sequence in SRP‐dependent PEX3 targeting to and integration into the ER membrane

**Figure 2:**
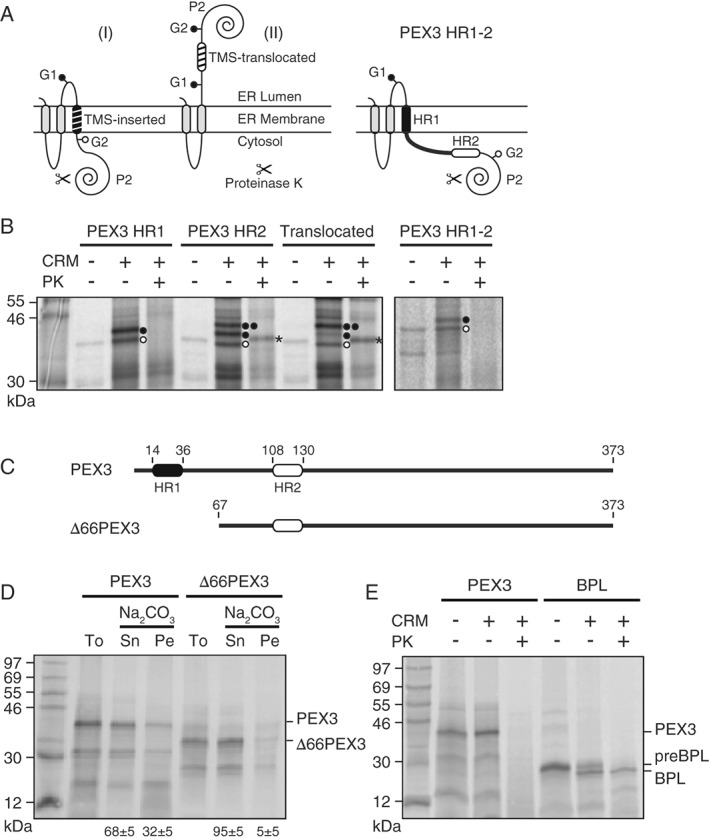
**HR1 is responsible for ER membrane insertion of PEX3**. A) Schematic representation of E. coli leader peptidase (Lep) constructs. A putative TMS (shaded) is engineered into the P2 domain flanked by two glycosylation acceptor sites (G1 and G2). Membrane integration of the TMS prevents enzymatic glycosylation of G2 on the lumenal side of the membrane (I), whereas both sites are glycosylated when a TMS does not insert into the membrane (II). In the latter case, ER‐lumenal P2 is also protected from Proteinase K (PK) treatment. The model of a Lep construct containing both HRs of PEX3 (PEX3 HR1‐2) suggests that only HR1 is inserted into the membrane. B) Insertion of PEX3 HR1 (residues 14–36), HR2 (residues 108–130), or HR1‐HR2 (residues 14–130) fragments into the ER bilayer. PEX3‐HR‐Lep chimeras or a translocated control (construct no. 67; 58) were translated in RRL in either the presence or absence of column‐washed rough microsomes (CRM). [^35^S]Met‐labeled proteins were analyzed directly or treated with PK. Unglycosylated (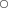), mono‐ (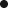), double‐glycosylated (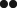), and P2‐containing protease‐protected fragments (*) are indicated. C) Scheme of full‐length and truncated PEX3 lacking the N‐terminal 66 residues (Δ66PEX3). D) Full‐length PEX3 is anchored in the ER bilayer. [^35^S]Met‐PEX3 was translated in RRL supplemented with CRM, and products were subjected to sodium carbonate extraction at pH 11.5 and separated by centrifugation. The supernatant (Sn), the membrane pellet (Pe) and an untreated aliquot (To) are shown. Numbers indicate the average amount of PEX3 or Δ66PEX3 in the supernatant and membrane pellet fractions, respectively. The averages ± SD of at least three independent experiments are shown. E) Orientation of ER‐inserted PEX3. Full‐length PEX3 or secreted bovine prolactin (BPL) was translated as above in either the absence or presence of CRM. Translation products were analyzed directly or treated with PK. pre, BPL with an uncleaved signal sequence.

The HR1 interaction with SRP indicates that the nonpolar HR1 acts as a signal sequence. Does HR1 also function as a transmembrane segment (TMS) to anchor PEX3 in the membrane? PEX3 HR segments were engineered into the *Escherichia coli* inner membrane protein leader peptidase 57 (Figure 2A), and the glycosylation pattern revealed that isolated HR1, but not HR2, was efficiently integrated into the ER membrane (Figure 2B). Moreover, single‐glycosylation of a Lep‐derived chimera that contained both HRs (connected by their natural‐occurring linker sequence) suggests that only one bilayer‐spanning segment (HR1) exists within the HR1‐HR2 fragment of PEX3 (Figure 2B). Furthermore, carbonate extraction of PEX3 and a derivative lacking HR1 (Figure 2C) showed that HR1 is necessary (Figure 2D) and sufficient (Figure S3) for stable insertion of PEX3 into the ER bilayer. Finally, protease sensitivity revealed that the large C‐terminal domain of ER‐inserted PEX3 is exposed to the cytosol (Figure 2E), a topology previously described for peroxisomal‐localized PEX3 51, 59. Since ER membrane‐integrated and non‐inserted PEX3 had identical molecular masses (Figure 2D,E), the N‐terminal hydrophobic HR1 of PEX3 acts as a non‐cleavable signal‐anchor TMS that is recognized by SRP.

**Figure 3:**
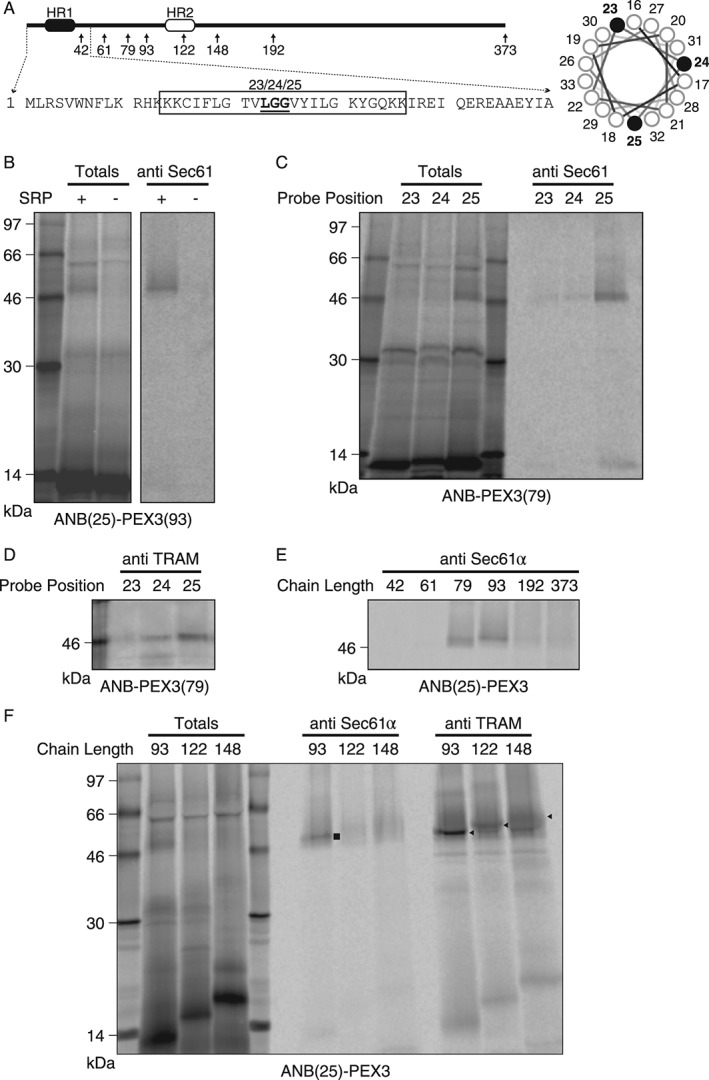
**Photocrosslinking of nascent PEX3 to the translocon proteins Sec61α and TRAM.** A) Scheme and N‐terminal sequence of PEX3. Arrows indicate nascent chains of different lengths. An amber stop codon was substituted at position L23, G24 or G25 (underlined) to position the photoreactive ANB at a single nascent chain location within HR1 (boxed). Probes project from different sides of the TMS α‐helix surface as shown in the helical wheel projection (right). B) Photocrosslinking to Sec61α is SRP‐dependent. [^35^S]Met‐ANB(25)‐PEX3(93) nascent chains were prepared in wheat germ extract supplemented with canine CRM in either the absence or presence of canine SRP. Photoadducts were analyzed directly (Totals, 1/20 aliquot) or after immunoprecipitation with antibodies specific for Sec61α. C–F) Photocrosslinking to Sec61α and TRAM. [^35^S]Met‐labeled ANB(23)‐PEX3, ANB(24)‐PEX3 or ANB(25)‐PEX3 integration intermediates of different length were translated in the presence of CRM and SRP. Photoadducts were analyzed either directly (Totals, 1/20 aliquot) or after immunoprecipitation with antibodies directed against Sec61α or TRAM, respectively. Photoadducts containing Sec61α (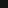) or TRAM (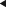) are indicated in (F). Uncropped images of (D) and (E) are shown in Figure S5.

Following SRP•RNC docking at the ER membrane, the Sec61 translocon mediates both the transport of soluble proteins into the ER lumen and the insertion of integral membrane proteins laterally into the ER bilayer 38. The mammalian Sec61 translocon is composed of four core proteins, Sec61α,β,γ and the translocating chain‐associating membrane protein (TRAM; 48). To examine SRP dependence of PEX3 targeting to the translocon, PEX3‐RNCs were translated in a wheat germ extract that has such a low endogenous content of SRP that RNC targeting to canine column‐washed rough microsomes (CRM) is dependent on added canine SRP 55, 60. ANB(25)‐PEX3(93) RNCs were prepared in the presence of CRM, and either the presence or absence of SRP. After photolysis and immunoprecipitation using antibodies specific for Sec61α, covalent photoadducts between Sec61α and PEX3 nascent chains were observed only in the presence of SRP (Figure 3B). No photoadducts were observed in the absence of the photoreactive probe (data not shown). Thus, SRP is required to target nascent PEX3 to the translocon.

### PEX3 interacts with translocon proteins Sec61α and TRAM in a defined and ordered multistep sequence

To further characterize PEX3 HR1 interactions at the ER translocon, we used a high‐resolution photocrosslinking approach. Parallel samples of same length ANB(23)‐PEX3(79), ANB(24)‐PEX3(79) and ANB(25)‐PEX3(79) integration intermediates were generated and photolyzed, and the extent of photocrosslinking to translocon proteins was determined by immunoprecipitation with antibodies specific for Sec61α and TRAM. The ANBs incorporated at three sequential residues within HR1 project from three different sides of the TMS α‐helix (Figure 3A). If HR1 is randomly oriented when it is proximal to Sec61α, then all three probes should react equally with Sec61α and/or TRAM. However, if an asymmetric photocrosslinking pattern is observed, then HR1 must be held in a fixed orientation adjacent to Sec61α and/or TRAM 54. Since only probes at residue 25 of PEX3 photocrosslinked to Sec61α (Figure 3C), probes at both positions 24 and 25 photocrosslinked to TRAM (Figure 3D), and probes at residue 23 photocrosslinked to neither translocon protein, the asymmetry of photocrosslinking reveals that HR1 is bound and held at a specific site within the translocon.

HR1 proximity to translocon proteins was then examined as a function of nascent chain length. Since an ϵANB‐Lys at PEX3 residue 25 photocrosslinked to both Sec61α and TRAM, ANB(25)‐PEX3 RNCs with increasing nascent chain lengths were prepared in parallel, photolyzed and analyzed by immunoprecipitation. When nascent chain length increased beyond 93 residues, HR1 was no longer adjacent to Sec61α (Figure 3E,F). TRAM‐containing photoadducts were observed with nascent chain lengths of 93 and 122, 148 to a lesser extent (Figure 3F), and not at all for nascent chains 192 or more residues (Figure S4). Since HR1 was adjacent to TRAM, but not to Sec61α, at 122 residues, HR1 was retained next to TRAM longer than to Sec61α, consistent with earlier data showing a TMS passing sequentially from Sec61α to TRAM during integration at the translocon 61, 62. Human PEX3 therefore inserts co‐translationally into the ER membrane via a SRP‐dependent and defined translocon‐mediated multistep pathway.

In yeast, the only peroxin mRNA that co‐localized at the ER was that of PEX3 63, a result indirectly suggesting that the Sec61p translocon facilitates the co‐translational insertion of PEX3 into the yeast ER. Other recent studies support the involvement of the yeast Sec61p translocon in PMP integration 20, 21, whereas previous reports 64 came to the opposite conclusion. By taking all differences in the experimental setups into account, there is now an increasing appreciation that the yeast Sec61p translocon is required for PMP insertion into the yeast ER (reviewed in 31 32, 65 66). However, several key issues remain unresolved. It is not known how PMPs reach the translocon in yeast, or whether PMP insertion into the yeast ER occurs co‐ or post‐translationally 21. Similarly, it was not known whether the Sec61 translocon was required for PMP insertion into the ER in mammals. But here we show for the first time that a N‐terminal TMS of a nascent PMP is recognized by SRP as it emerges form the ribosome (Figure 1), and that SRP is required to target the PMP‐containing RNC to the translocon (Figure 3). These results are in line with recent data showing that the first TMS of PEX16 is necessary for its targeting to the ER(8). In addition, our data show that the nascent chain of the mammalian PMP PEX3 is co‐translationally inserted into the ER bilayer adjacent to the translocon proteins Sec61α and TRAM in a multistep process (Figure 3). These results therefore establish the Sec61 translocon as ER entry point for mammalian PMPs, as well as providing mechanistic details of PMP targeting to and insertion into the mammalian ER membrane.

Does every human PEX3 insert into the ER membrane via the SRP‐ and translocon‐mediated pathway? Given the sub‐stoichiometric number of SRPs relative to ribosomes (1–2 SRPs/100 yeast ribosomes 67, and 5–8 SRPs/100 mammalian ribosomes 68), it is certainly possible that PEX3 molecules may escape recognition by SRP and be inserted post‐translationally into peroxisomes 11 or the ER 25, 26. On the other hand, the co‐translationally inserted PEX3 in the ER may serve as docking factor for PEX19•cargoPMP complexes 10, 11 and thereby concentrate other PMPs or PMP sub‐complexes 30 in a spatially defined area of the ER. The initial co‐translational insertion of human PEX3 at a Sec61 translocon would therefore be a critical and essential step in seeding the mammalian ER with peroxins.

### PEX3 exits the ER via budded vesicles

**Figure 4:**
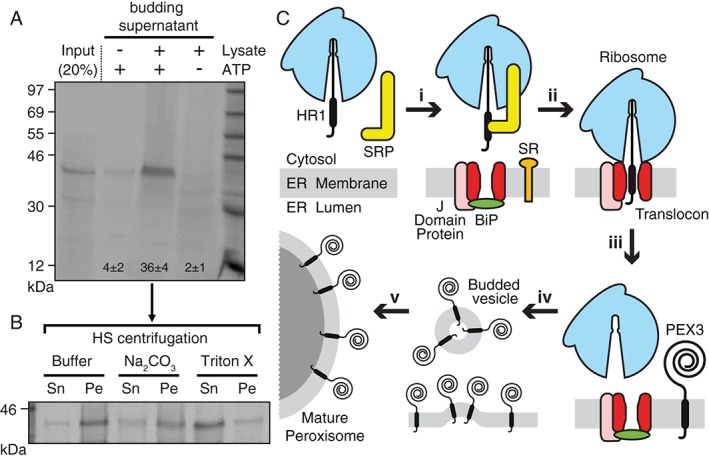
**Cell‐free vesicle budding of PEX3.** A) Full‐length [^35^S]Met‐PEX3 was transcribed/translated in RRL in the presence of CRM. Washed donor membranes were incubated at 30°C in the presence of either buffer A (− lysate) or RRL. Samples were either substituted with an ATP‐regenerating system (+ATP) or treated with apyrase (−ATP). After the budding reaction, donor membranes were removed by sedimentation, and the supernatant fraction and a 20% aliquot (Input) of the starting microsomes were analyzed. Numbers indicate the average amount of budded PEX3 ± SD for at least three independent experiments. B) The supernatant of an ATP‐ and lysate‐containing budding reaction was subjected to high‐speed (HS) centrifugation, and the pellet was resuspended in buffer A with or without 1% (v/v) Triton X‐100 or subjected to 0.1 m sodium carbonate extraction at pH 11.5. After a second centrifugation step, the protein contents of the supernatant (Sn) and pellet (Pe) fractions were analyzed. C) Model of human PEX3 passage through the ER. During ribosomal translation of PEX3, HR1 is recognized and bound by SRP (i). After SRP‐dependent targeting of the RNC to the ER membrane (ii) via the SRP receptor (SR), PEX3 is co‐translationally integrated into the mammalian ER at the Sec61 translocon and its associated proteins (J Domain Protein, BiP; 69) (iii). Following integration into the ER membrane, PEX3 is selectively packed into budding vesicles in an ATP‐ and cytosol‐dependent process (iv). PEX3‐containing budded vesicles then either fuse with pre‐existing peroxisomes or initiate peroxisomal de novo synthesis (v).

Is PEX3 integration into the ER membrane a precursor to PEX3 transport to the peroxisome? If so, one would predict that PEX3 is segregated into specific regions of the ER membrane for budding and transport to the peroxisome 27. A cell‐free vesicle budding assay recently established in yeast 28, 29 shows that PMP‐containing carrier vesicles are released from the ER in a cytosol‐ and ATP‐dependent process. To determine whether human PEX3 is packed into vesicles that bud from mammalian ER membranes, full‐length PEX3 was translated in vitro in the presence of canine ER microsomes. Following translation, membranes were collected and washed extensively to remove any peripherally attached PEX3. These microsomes were then used as donor membranes to study the ER exit of PEX3 in the presence of rabbit reticulocyte lysate (RRL), ATP and an ATP‐regenerating system. After the budding reaction, the larger and more dense donor microsomal membranes were removed by medium‐speed centrifugation. PEX3 was then detected in the supernatant fraction of samples containing RRL and ATP, but not in the supernatant of samples lacking either cytosol or ATP (Figure 4A). Budded PEX3 could be collected by high‐speed centrifugation, was resistant to carbonate extraction, and was solubilized in detergent (Figure 4B), thereby indicating that PEX3 was localized in a membrane of small vesicles. Since 36 ± 4% of the total integrated PEX3 was recovered in the supernatant in the presence of cytosol and ATP (Figure 4A), PEX3 was apparently selected and preferentially transferred to the small ER‐derived vesicles.

Great attention has been paid to the mechanisms involved in the vesicular trafficking of PMPs from ER to peroxisomes in yeast. Recent studies revealed that new peroxisomes are formed via heterotypic fusion of at least two biochemically distinct preperoxisomal vesicle pools that arise from the ER 30. However, the detailed molecular basis for the budding of these preperoxisomal structures in yeast remains unclear 70. In mammals, it was unknown whether small ER‐derived vesicles play a role in the mammalian peroxisomal *de novo* biogenesis. But our data now provide the first direct evidence that human PMPs are actively and selectively extracted from mammalian ER membranes in a cytosol‐dependent and ATP‐consuming vesicle budding reaction. As previously reported in yeast 19, 28, 29, 30, these data are consistent with small ER‐derived vesicles playing a role in PEX3 trafficking to mammalian peroxisomes. By establishing a mammalian cell‐free budding assay, we provide a new experimental platform that can both examine the precise distribution and binding partners of newly inserted PMPs in the ER, and identify the components in the cytosol that are involved in the budding reaction. Such information is crucial for understanding the *de novo* formation of peroxisomes from the ER in mammals.

The combined data presented here establish that nascent human PEX3 is targeted to the mammalian ER membrane by SRP, integrates co‐translationally at the mammalian translocon, and then is selectively packaged and extracted from the ER membrane via an energy‐ and cytosol‐dependent budding reaction. By experimentally characterizing the entire pathway required for PEX3 passage through the ER (Figure 4C), the transient role of the ER in mammalian peroxisomal biogenesis has now been demonstrated from recruitment and entry to exit and discharge. On the other hand, it has been reported that certain integral PMPs, including PEX3 11, can insert post‐translationally into mature peroxisomes 6, 10, 35. The existence of these two distinct pathways, the co‐translational insertion into the ER via the Sec61 translocon as detailed in this study, and the post‐translational insertion into mature peroxisomes 6, 10, 11, 35, is in good agreement with the currently widely accepted semiautonomous model of peroxisomal biogenesis 3, 31, 32, 33, 34. According to this working model, a dynamic peroxisomal homeostasis is ensured by both the recruitment of PMP‐containing membranes from the ER via budded vesicles and the enhanced accumulation of PMP and matrix proteins in pre‐existing peroxisomes, thereby facilitating fast peroxisomal propagation by growth and division. Since differences in the relative contribution of these two routes are likely to depend on the organism or its cellular conditions, a future challenging goal is to determine what fraction of PMPs, and particularly PEX3 molecules, is inserted directly into pre‐existing peroxisomes instead of transiting through the ER. While the mechanisms that regulate when, where and how a PMP will follow a particular route are currently unknown, the data herein show that co‐translational mammalian PEX3 targeting to and insertion into the ER membrane occurs via SRP and the Sec61 translocon, and that PEX3 exit from the mammalian ER occurs via budded vesicles in an ATP‐dependent process. By establishing the mechanisms of PEX3 entry into and exit from the mammalian ER, the regulation of PEX3 trafficking can now be addressed and quantified directly.

## Materials and Methods

### Plasmids, mRNA, tRNA, SRP and microsomes

All PEX3 constructs originated from the plasmid pcDNA3.1/PEX3mychis that encodes the human full‐length PEX3, as previously described 51. The introduction of a single amber stop codon at selected locations was done using the Quikchange protocol (Agilent Technologies). Bovine prolactin is encoded in the plasmid pSP64‐BPL 71. For the membrane insertion of isolated PEX3‐segments, HR1 (residues 14–36), HR2 (residues 108–130) or HR1‐HR2 (residues 14–130) fragments were independently amplified and introduced into the modified *E. coli* leader peptidase (Lep) sequence from the pGEM1 plasmid 58 using the *Spe*I/*Kpn*I sites. The primary sequence of each construct was confirmed by DNA sequencing. mRNA was transcribed *in vitro* using SP6 RNA polymerase and PCR‐generated DNA fragments of the desired length as before 54. Reverse primers either contained an ochre stop codon to obtain full‐length PEX3 translation products (e.g. for the budding assay) or lacked a stop codon for the generation of RNCs. Primer sequences are available from the authors on request. [^14^C]Lys‐tRNA^amb^, ϵANB‐[^14^C]Lys‐tRNA^amb^, ϵNBD‐[^14^C]Lys‐tRNA^amb^, canine CRM and purified SRP from dog pancreas in SRP buffer [50 mm triethanolamine (pH 7.5), 600 mm KOAc (pH 7.5), 6 mm Mg(OAc)_2_, 1 mm DTT] were obtained from tRNA Probes. SRP concentration was determined using ϵ_280nm_ = 1.0 × 10^6^ M^−1^cm^−1^.

### Cell‐free translation in RRL

*In vitro* translation of purified mRNA (typically 25 μL, 30°C, 40 min) was performed in the presence of RRL (Promega), [^35^S]Met (0.4 μCi/μL), and, when indicated, 4 equivalents (eq., 72) CRM. After translation, samples were either analyzed directly by SDS‐PAGE and phosphorimaging (PharosFX molecular imager, Bio‐Rad), or membranes were collected by sedimentation (Beckman TLA100 rotor; 430 000 × g; 5 min; 4°C) through a 0.5 m sucrose cushion in buffer A [30 mm HEPES (pH 7.5), 120 mm KOAc, 3.2 mm Mg(OAc)_2_]. For proteolysis experiments (Figure 2E), samples were treated with 200 µg/mL proteinase K for 30 min on ice followed by the addition of 1 mm phenylmethylsulfonyl fluoride. For carbonate extraction 73, membranes were incubated in carbonate buffer [0.1 m Na_2_CO_3_ (pH 11.5)] for 15 min on ice, centrifuged (Beckman TLA100 rotor; 430 000 × g; 5 min; 4°C), washed and resuspended in carbonate buffer. The supernatant and pellet fraction were neutralized with glacial acetic acid and further analyzed as above.

Lep‐derived constructs were transcribed and translated in the presence of RRL, [^35^S]Met and canine CRM as described previously 74. Samples were analyzed by SDS‐PAGE, and visualized on a Fuji FLA3000 phosphorimager using image gauge software. The proteinase K digestions were performed after *in vitro* translation by incubation the mixture with 400 µg/mL proteinase K on ice for 40 min (Figure 2B). The reaction was stopped by adding 2 mm phenylmethylsulfonyl fluoride. The membrane fraction was then collected by centrifugation and analyzed by SDS‐PAGE.

### Photocrosslinking and immunoprecipitation

*In vitro* translations (typically 50 μL, 26°C, 40 min) of truncated mRNAs were performed in wheat germ cell‐free extract (tRNA Probes) in the presence of 40 nm canine SRP, 8 eq. CRM, [^35^S]Met (1.0 μCi/μL), 0.6 pmol/μL [^14^C]Lys‐tRNA^amb^/ϵANB‐[^14^C]Lys‐tRNA^amb^ as indicated, and other components as described 52. Samples were photolyzed on ice for 15 min using a 500 W mercury arc lamp 54. After photolysis, samples were collected by sedimentation (5 min for CRM or 60 min for free RNCs) through a 0.5 m sucrose cushion in buffer A as described above. Pellets were resuspended in 3% (w/v) SDS and 50 mm Tris–HCl (pH 7.5), then incubated at 55°C for 30 min. Samples were brought up to 500 μL with either buffer S [140 mm NaCl, 10 mm Tris–HCl (pH 7.5), and 2% (v/v) Triton X‐100] for Sec61α‐specific antibodies, or buffer T [150 mm NaCl, 1 mm EDTA, 50 mm Tris–HCl (pH 7.5), 1% (v/v) Triton X‐100] for TRAM‐ or SRP54‐specific antibodies. Samples were precleared by rocking with protein A‐Sepharose (Sigma‐Aldrich; 40 μL; pre‐equilibrated in buffer S or T) at 4°C for 1 h. After removal of the beads by centrifugation, the supernatants were incubated overnight at 4°C with affinity‐purified rabbit antisera specific either for Sec61α or TRAM 54, or for SRP54 (BD Biosciences). Protein A‐Sepharose (40 μL, pre‐equilibrated with buffer S or T) was then added and incubated for 4 h at 4°C. Sepharose beads were harvested by sedimentation and washed twice with 750 μL of buffer S or T, followed by a final washing in the same buffer without detergent. Samples were then analyzed by SDS‐PAGE and phosphorimaging.

### Fluorescence spectroscopy

*In vitro* translations (500 μL total volume, 26°C, 40 min) of truncated mRNAs were performed in wheat germ cell‐free extract in the presence of 0.6 pmol/μL ϵNBD‐[^14^C]Lys‐tRNA^amb^ and other components as described 52. To correct for the significant background signal due to light scattering from the ribosomes, equivalent blank translation reactions lacking NBD were prepared in parallel with [^14^C]Lys‐tRNA^amb^. RNCs were purified by gel filtration at 4°C using a Sepharose CL‐6B column (1.5 cm inner diameter × 20 cm) and buffer A as elution buffer. A slow flow rate was used during gel filtration to ensure the removal of noncovalently bound fluorophores. The absorbance at 260 nm of each 550 μL fraction was used to identify those fractions containing RNCs that elute in the void volume, and only the leading half of the void volume peak was pooled. After gel filtration, the absorbance at 260 nm of the two parallel samples (one with and one without NBD) was equalized before initiating spectral measurements. Steady‐state fluorescence measurements were made with either an SLM‐8100 or a Spex Fluorolog‐3 spectrofluorometer at 4°C as described previously 53. Samples (250 μL) were placed in 4 × 4 mm quartz microcells that were coated with phosphatidylcholine vesicles to minimize protein adsorption 75. The cuvette chamber was continuously flushed with N_2_ to prevent condensation of water on the microcells. Emission intensity (*λ*_ex_ = 468 nm) was scanned at 1‐nm intervals between 500 and 580 nm. Samples of purified RNCs with or without NBD in buffer A were titrated at 4°C by the sequential addition of known amounts of SRP in small volumes. After each addition, the emission intensities of the NBD and blank samples were measured after reaching equilibrium. After blank subtraction and dilution correction, the observed change in net NBD emission intensity (ΔF; *λ*_ex_ = 468 nm; *λ*_em_ = 528 nm, bandpass 4 nm) at each point in the titration was compared with the initial intensity (*F*_0_) of the sample in the absence of SRP.

### Budding assay

Purified full‐length PEX3 mRNA was translated in RRL in the presence ER microsomes as described above. The translation (60 min, 30°C) was stopped by addition of puromycin (2 mm final, 20 min, 4°C) and microsomes were collected by centrifugation through a 0.5 m sucrose cushion in buffer A as above. Membranes were incubated in 2.5 m urea in buffer A for 10 min at 4°C to remove peripherally bound PEX3 molecules. Membranes were collected by medium‐speed centrifugation (20 000 × ***g***, 10 min, 4°C), washed once in urea buffer, and finally washed in buffer A. Such PEX3 containing donor membranes were resuspended in buffer A, and incubated with either RRL (the lysate was diluted to 60% of its original concentration in the budding reaction) or an equivalent amount of buffer A. Budding reactions also contained 2 mm puromycin and either an energy generating system (final concentrations: 16 mm phosphocreatine, 2 mm ATP, 2 mm GTP, 0.016 U/μL phosphocreatine kinase) or 1 U/μL apyrase. After incubation of the budding reaction for 60 min at 30°C, donor membranes were removed by medium‐speed centrifugation, and the supernatant was analyzed by SDS‐PAGE and phosphorimaging. In certain cases, the supernatant of a budding reaction was further subjected to high‐speed centrifugation (Beckman TLA100 rotor; 55 000 rpm; 30 min; 4°C), and the pellet was resuspended in either carbonate buffer or 0.25 m sucrose in buffer A in the presence or absence of 1% (v/v) Triton X‐100. After a second high‐speed centrifugation, the protein content of the supernatant and pellet fractions was analyzed as above.

## Supporting information


**Figure S1: Photocrosslinking of PEX3(61)‐ and PEX3(93)‐RNCs to SRP.** [^35^S]Met‐ANB(25)‐PEX3‐RNCs were photolyzed and then analyzed by SDS‐PAGE and phosphorimaging either directly (Totals, 1/20 aliquot) or after immunoprecipitation with antibodies directed against SRP54. Photoadducts containing SRP54 (⧫) are indicated.
**Figure S2: SRP storage buffer does not alter the emission intensity of fluorescence‐labeled PEX3.** Truncated PEX3^G25amb^ mRNA was translated in wheat germ extract in the presence of ϵNBD‐Lys‐tRNA^amb^. Emission scans (λ_ex_ = 468 nm) of purified NBD(25)‐PEX3(93)‐RNCs were performed in buffer A before (−SRP buffer) and immediately after the addition of SRP storage buffer (+SRP buffer, equal volume as in Figure 1D).
**Figure S3: HR1 of PEX3 is stably anchored in the ER bilayer.** A) Schematic representation of full‐length PEX3 and a C‐terminally truncated PEX3 variant of 79 residues length (PEX[79]). Two predicted hydrophobic α‐helical regions (HR) are indicated by black (HR1) and white (HR2) boxes. B) PEX[79] was translated in rabbit reticulocyte lysate in the presence of CRMs. [^35^S]Met‐labeled translation products were subjected to sodium carbonate extraction at pH 11.5. After centrifugation (100 000 × **g**; 20 min), the supernatant (Sn) and the membrane pellet (Pe) were analyzed by SDS‐PAGE and visualized by phosphorimaging.
**Figure S4: Photocrosslinking of PEX3 to TRAM depends on nascent chain length.** [^35^S]Met‐labeled integration intermediates containing ANB(25)‐PEX3 nascent chains were prepared in parallel in wheat germ extract (supplemented with canine ER microsomal membranes and 40 nm canine SRP) with lengths of 42, 61, 79, 93, 192 and 373 (full‐length) residues. After photolysis, photoadducts were immunoprecipitated with antibodies directed against TRAM and analyzed by SDS‐PAGE and phosphorimaging.
**Figure S5: Uncropped phosphorimager scans of Figure** 3 **D,E.**
