# Supplementary material for: Human Peroxin PEX3 Is Co‐translationally Integrated into the ER and Exits the ER in Budding Vesicles
Source: Traffic. 2015 Dec 21;17(2):117–30. doi: 10.1111/tra.12350 (PMC5064655; doi:10.1111/tra.12350)
Supplement: Supplementary file 1 — Figure S1: Photocrosslinking of PEX3(61)‐ and PEX3(93)‐RNCs to SRP. [35S]Met‐ANB(25)‐PEX3‐RNCs were photolyzed and then analyzed by SDS‐PAGE and phosphorimaging either directly (Totals, 1/20 aliquot) or after immunoprecipitation with antibodies directed against SRP54. Photoadducts containing SRP54 (⧫) are indicated. Figure S2: SRP storage buffer does not alter the emission intensity of fluorescence‐labeled PEX3. Truncated PEX3G25amb mRNA was translated in wheat germ extract in the presence of ϵNBD‐Lys‐tRNAamb. Emission scans (λ ex = 468 nm) of purified NBD(25)‐PEX3(93)‐RNCs were performed in buffer A before (−SRP buffer) and immediately after the addition of SRP storage buffer (+SRP buffer, equal volume as in Figure 1D). Figure S3: HR1 of PEX3 is stably anchored in the ER bilayer. A) Schematic representation of full‐length PEX3 and a C‐terminally truncated PEX3 variant of 79 residues length (PEX[79]). Two predicted hydrophobic α‐helical regions (HR) are indicated by black (HR1) and white (HR2) boxes. B) PEX[79] was translated in rabbit reticulocyte lysate in the presence of CRMs. [35S]Met‐labeled translation products were subjected to sodium carbonate extraction at pH 11.5. After centrifugation (100 000 × g; 20 min), the supernatant (Sn) and the membrane pellet (Pe) were analyzed by SDS‐PAGE and visualized by phosphorimaging. Figure S4: Photocrosslinking of PEX3 to TRAM depends on nascent chain length. [35S]Met‐labeled integration intermediates containing ANB(25)‐PEX3 nascent chains were prepared in parallel in wheat germ extract (supplemented with canine ER microsomal membranes and 40 nm canine SRP) with lengths of 42, 61, 79, 93, 192 and 373 (full‐length) residues. After photolysis, photoadducts were immunoprecipitated with antibodies directed against TRAM and analyzed by SDS‐PAGE and phosphorimaging. Figure S5: Uncropped phosphorimager scans of Figure 3 D,E. [file TRA-17-117-s001.docx]

**Human peroxin PEX3 is co-translationally integrated into the ER and exits the ER in budding vesicles**

**Peter U. Mayerhofer^1,2,5,^*, Manuel Bañó-Polo^3^, Ismael Mingarro^3^, and Arthur E. Johnson^1,4,^***

^1^Department of Molecular and Cellular Medicine, Texas A&M Health Science Center, 440 Reynolds Medical Building, College Station, TX 77843, USA

^2^Institute of Biochemistry, Biocenter, Goethe University Frankfurt, Max-von-Laue Str. 9, 60438 Frankfurt, Germany

^3^Departament de Bioquimica i Biologia Molecular, Universitat de Valencia, C/ Dr. Moliner, 50, E-46100 Burjassot, Spain

^4^Departments of Chemistry and of Biochemistry and Biophysics, Texas A&M University, College Station, TX 77843, USA

^5^Present address: School of Biosciences & Medicine, University of Surrey, Guildford, GU2 7XH, United Kingdom

*Co-Corresponding authors:

Dr. Peter U. Mayerhofer

Institute of Biochemistry Fax: +49-(0)-69-798-29495

Biocenter N210 Phone: +49-(0)-69-798-29475

Goethe University Frankfurt

Max-von-Laue Str. 9

60438 Frankfurt, Germany

e-mail: mayerhofer@em.uni-frankfurt.de; p.mayerhofer@surrey.ac.uk

Dr. Arthur E. Johnson

Department of Molecular and Cellular Medicine Fax: +1-979-847-9481

Texas A&M Health Science Center Phone: +1-979-862-3440

440 Reynolds Medical Building

College Station, TX 77843-1114, USA

e-mail: ajohnson@medicine.tamhsc.edu

**Supplemental Materials**


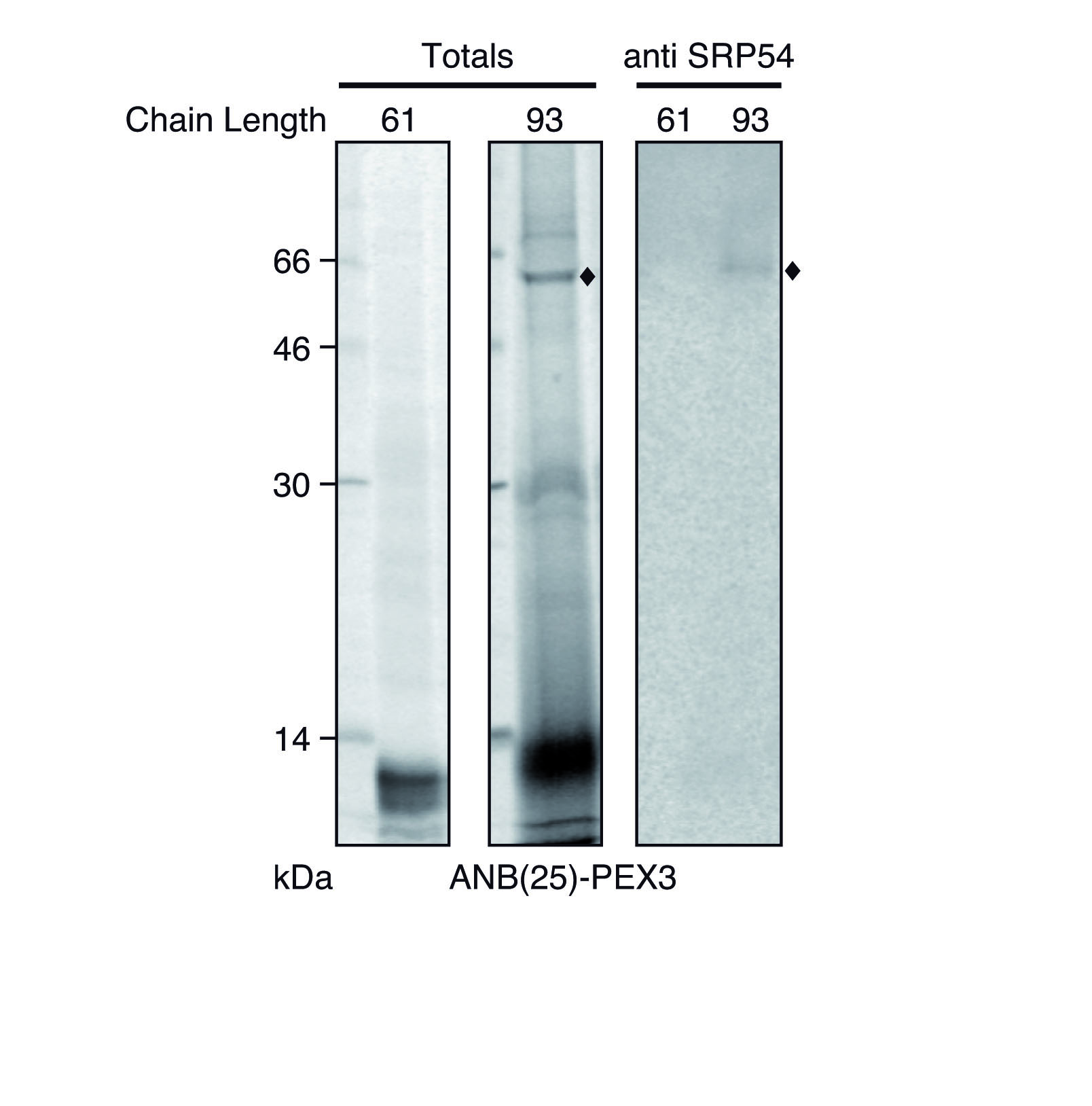


**Figure S1: Photocrosslinking of PEX3(61)- and PEX3(93)-RNCs to SRP.** [^35^S]Met-ANB(25)-PEX3-RNCs were photolyzed and then analyzed by SDS-PAGE and phosphorimaging either directly (Totals, 1/20 aliquot) or after immunoprecipitation with antibodies directed against SRP54. Photoadducts containing SRP54 (♦) are indicated.

**
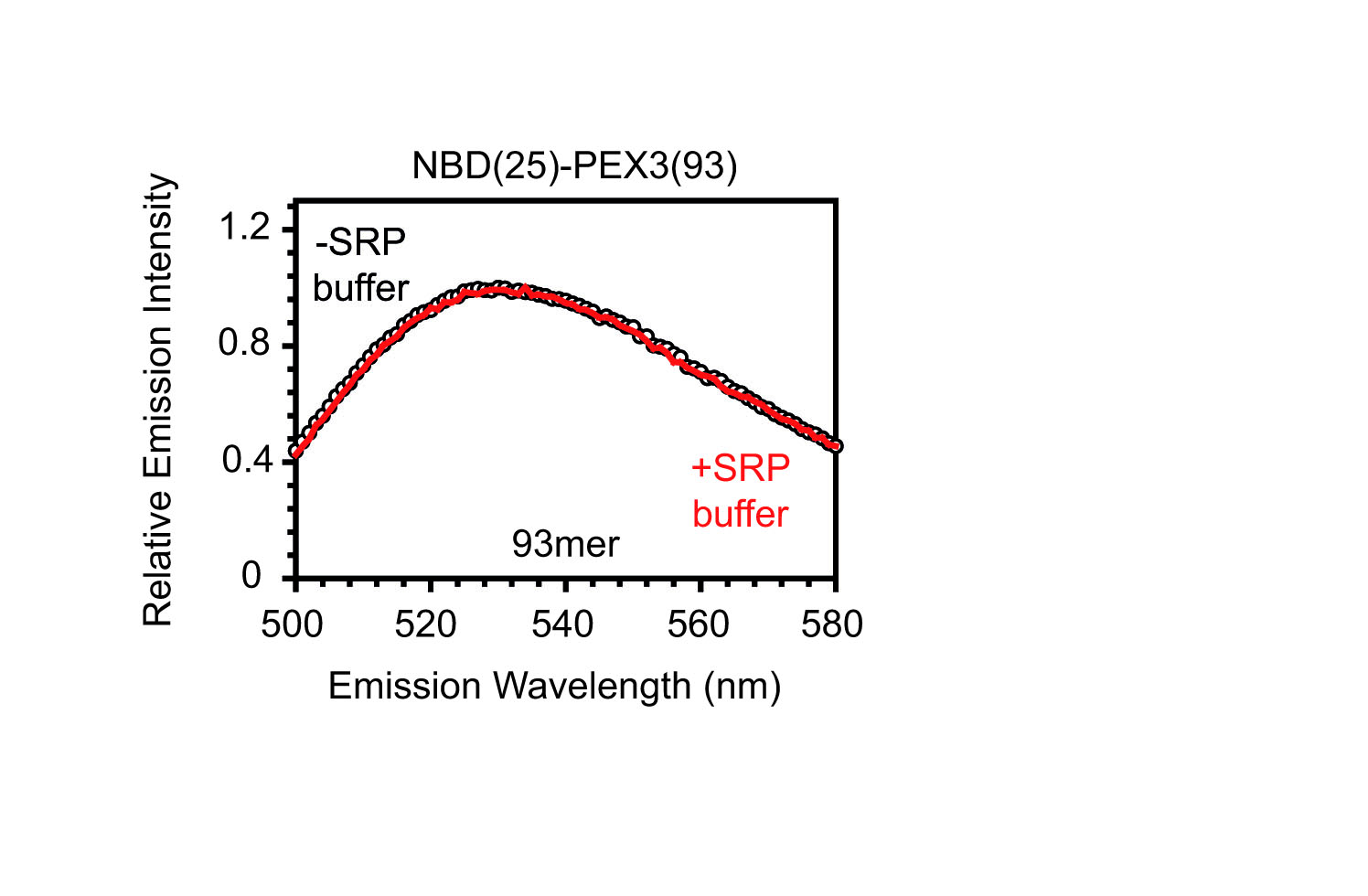
**

**Figure S2: SRP storage buffer does not alter the emission intensity of fluorescence-labeled PEX3.** Truncated PEX3^G25amb^ mRNA was translated in wheat germ extract in the presence of εNBD-Lys-tRNA^amb^. Emission scans (λ_ex_ = 468 nm) of purified NBD(25)-PEX3(93)-RNCs were performed in buffer A before (-SRP buffer) and immediately after the addition of SRP storage buffer (+SRP buffer, equal volume as in Figure 1D).

**
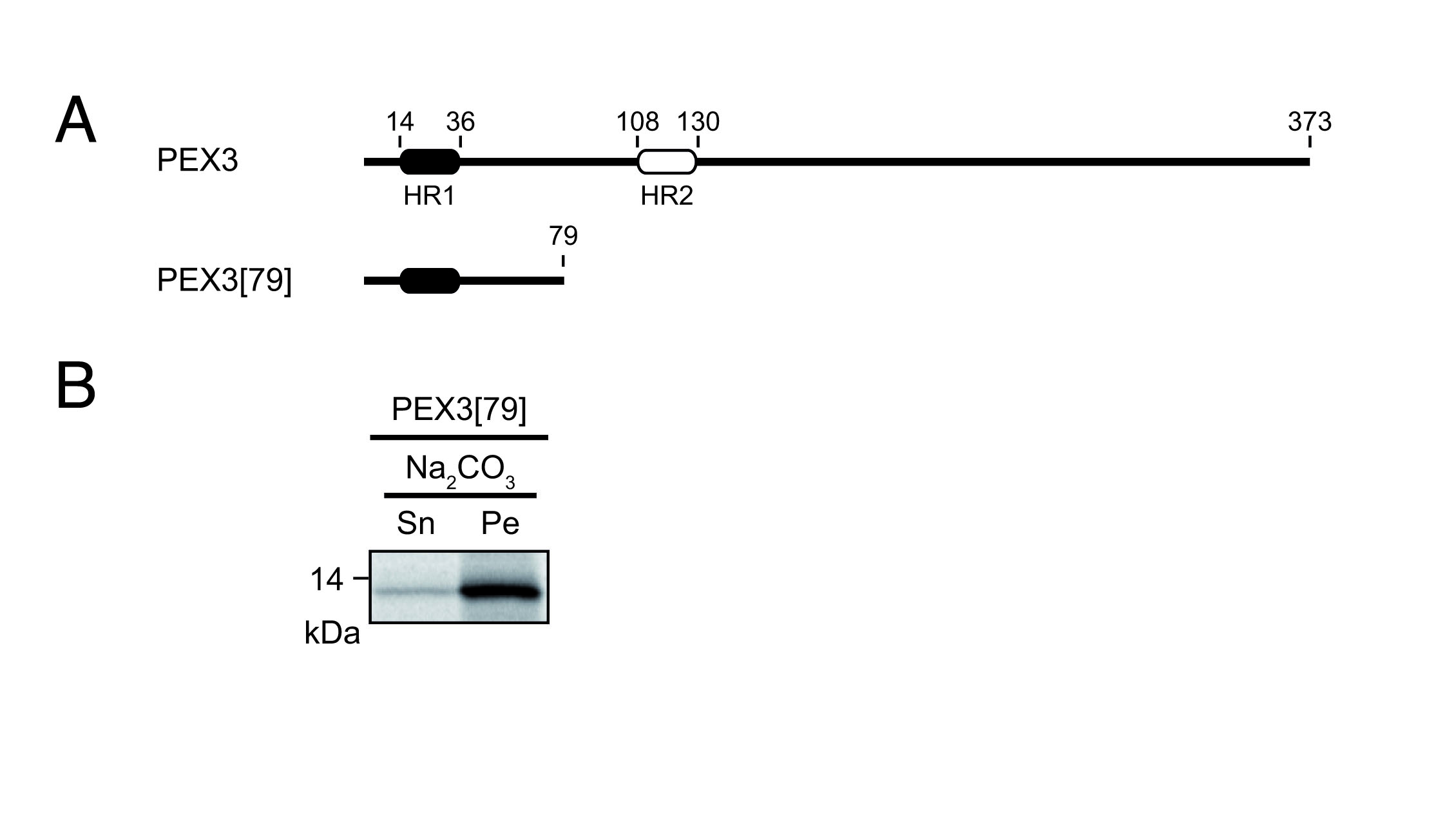
**

**Figure S3: HR1 of PEX3 is stably anchored in the ER bilayer.** A) Schematic representation of full-length PEX3 and a C-terminally truncated PEX3 variant of 79 residues length (PEX[79]). Two predicted hydrophobic α-helical regions (HR) are indicated by black (HR1) and white (HR2) boxes. B) PEX[79] was translated in rabbit reticulocyte lysate in the presence of CRMs. [^35^S]Met-labelled translation products were subjected to sodium carbonate extraction at pH 11.5. After centrifugation (100,000 x g; 20 min), the supernatant (Sn), and the membrane pellet (Pe) were analyzed by SDS-PAGE and visualized by phosphorimaging.

**
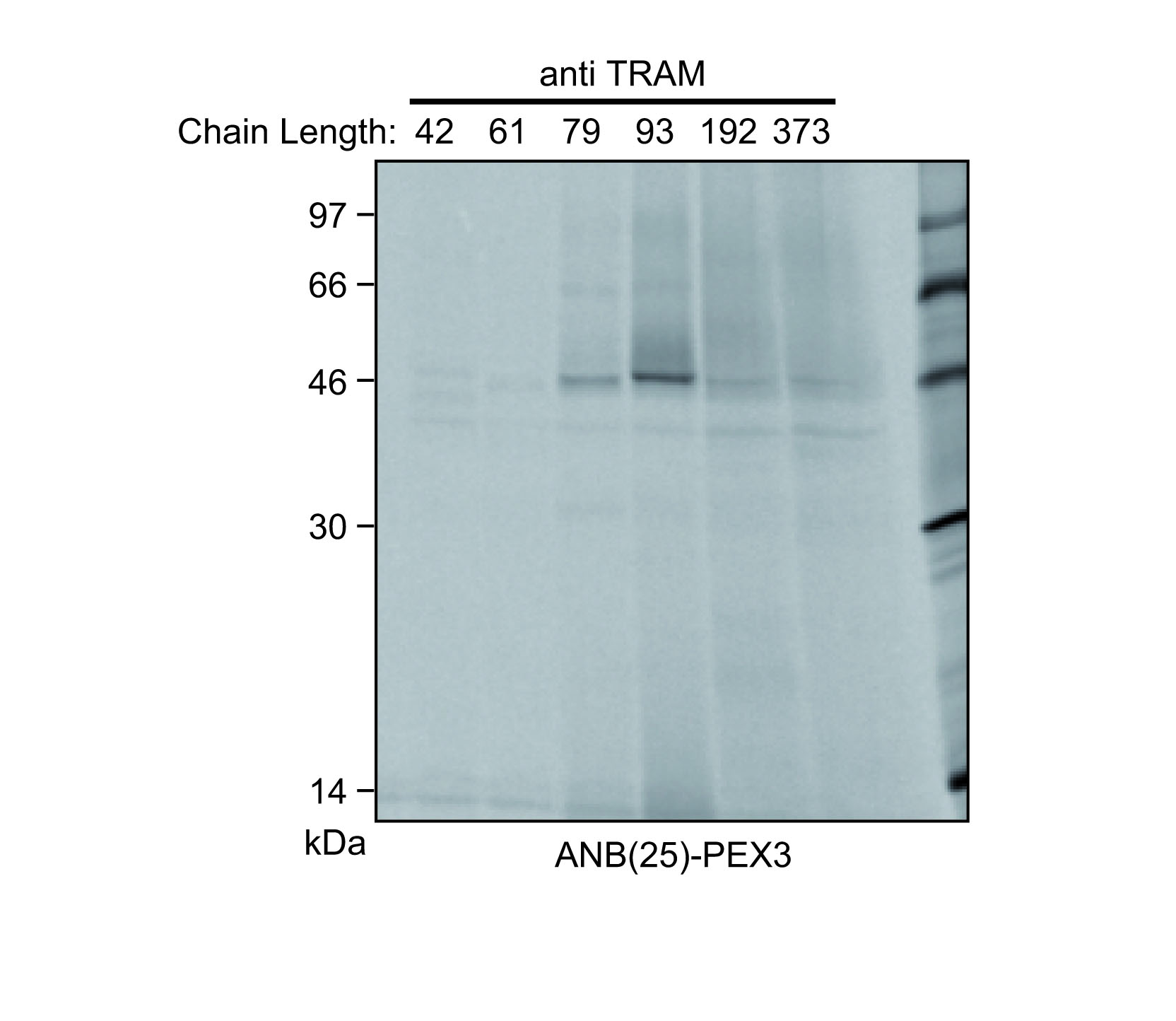
**

**Figure S4: Photocrosslinking of PEX3 to TRAM depends on nascent chain length.** [^35^S]Met-labelled integration intermediates containing ANB(25)-PEX3 nascent chains were prepared in parallel in wheat germ extract (supplemented with canine ER microsomal membranes and 40 nM canine SRP) with lengths of 42, 61, 79, 93, 192, and 373 (full-length) residues. After photolysis, photoadducts were immunoprecipitated with antibodies directed against TRAM and analyzed by SDS-PAGE and phosphorimaging.

**
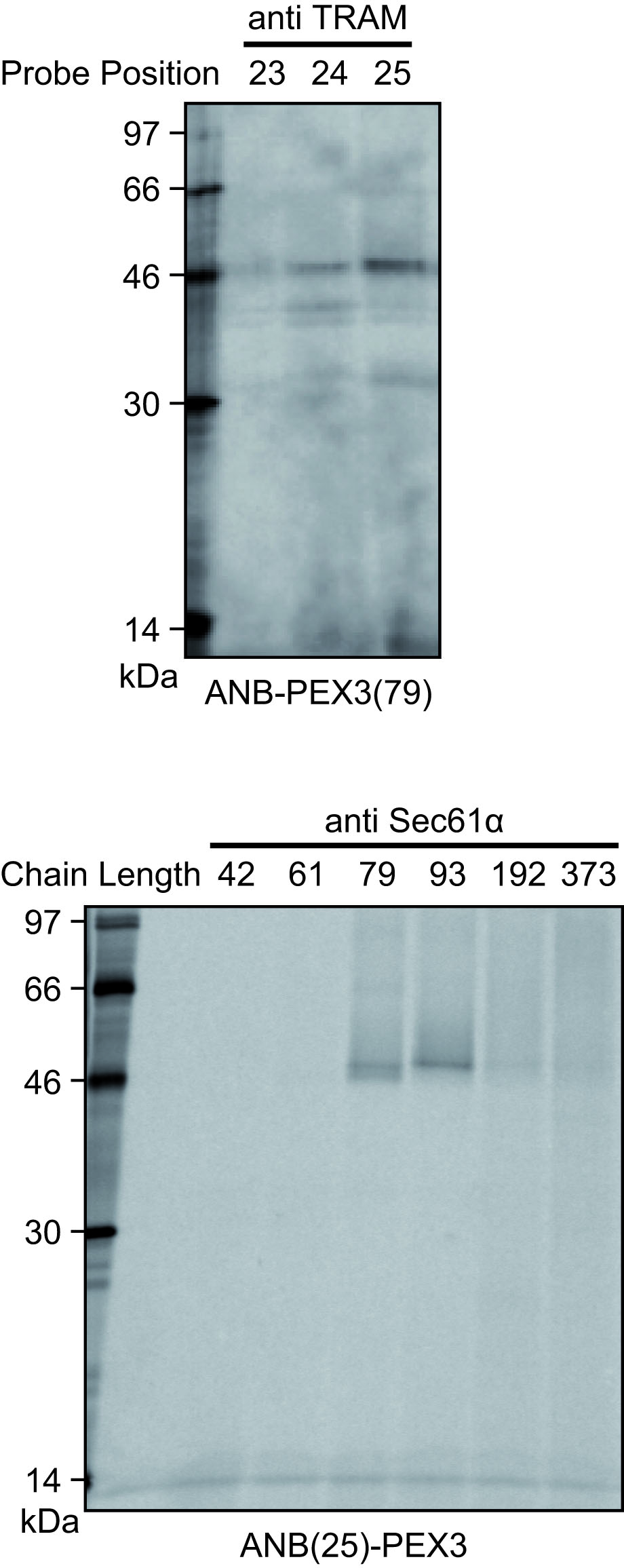
**

**Figure S5: Uncropped phosphorimager scans of Figures 3 D and 3 E.**
